# OOPS, the Ontology for Odor Perceptual Space: From Molecular Composition to Sensory Attributes of Odor Objects

**DOI:** 10.3390/molecules27227888

**Published:** 2022-11-15

**Authors:** Alice Roche, Nathalie Mejean Perrot, Thierry Thomas-Danguin

**Affiliations:** 1 Centre des Sciences du Goût et de l’Alimentation, INRAE, CNRS, Institut Agro, CNRS, Université Bourgogne Franche-Comté, F-21000 Dijon, France; 2 UMR MIA 518, AgroParisTech, INRAE, Université Paris Saclay, F-75015 Paris, France

**Keywords:** odor, perceptual space, odor quality, odor descriptor, odorant, wine, expert knowledge

## Abstract

When creating a flavor to elicit a specific odor object characterized by odor sensory attributes (OSA), expert perfumers or flavorists use mental combinations of odor qualities (OQ) such as Fruity, Green, and Smoky. However, OSA and OQ are not directly related to the molecular composition in terms of odorants that constitute the chemical stimuli supporting odor object perception because of the complex non-linear integration of odor mixtures within the olfactory system. Indeed, single odorants are described with odor descriptors (OD), which can be found in various databases. Although classifications and aroma wheels studied the relationships between OD and OQ, the results were highly dependent on the studied products. Nevertheless, ontologies have proven to be very useful in sharing concepts across applications in a generic way and to allow experts’ knowledge integration, implying non-linear cognitive processes. In this paper, we constructed the Ontology for Odor Perceptual Space (OOPS) to merge OD into a set of OQ best characterizing the odor, further translated into a set of OSA thanks to expert knowledge integration. Results showed that OOPS can help bridge molecular composition to odor perception and description, as demonstrated in the case of wines.

## 1. Introduction

Within the physical world, colors are characterized by light wavelength, tones by sound frequency, and odors by the chemical composition of the olfactory stimulus. Within the perceptual space, colors are defined by specific words such as “red” or “blue”, tones are referred to by dedicated notes such as “C” or “E♭”, and odors are usually identified by naming their sources such as “rose” or “lemon” [1]. Therefore, if colors and tones can be well defined experimentally through normative vocabulary, odors are difficult to describe with a consensual vocabulary. Odors are also difficult to measure physically because they mostly result from the coding, by the olfactory system, of complex mixtures of odorants, which are volatile organic compounds varying in chemical nature and concentration [2]. 

Olfactory coding induces perceptual interactions, which can take place at several steps of the olfactory information processing, and the odor perceived from mixtures of odorants is not a simple sum of the odors of each odorant embedded in the mixture [2]. Synergy and masking effects have been often reported [3,4,5,6], but also perceptual dominance [7], or configural and elemental perception [8,9]. For instance, a ternary mixture composed of three odorants, respectively, described as “strawberry”, “caramel”, and “violet”, elicits, at a specific proportion of each compound, the perception of a “pineapple” odor [10]. The mechanisms behind these perceptual interactions are not well understood yet and are still poorly investigated. As a consequence, the description of the perceptual outcome of a complex mixture using odor sensory attributes (OSA) is not straightforward. The global odor percept is especially hard to predict on the basis of the mixtures’ chemical composition, namely every single odorant that can be qualified with specific odor descriptors (OD).

Several databases compile the OD of large sets of odorants: Arctander’s Handbook [11], the Atlas of Odor Character Profiles [12], Fenaroli’s Handbook [13], Flavor-Base [14], Flavornet [15], Flavors and Fragrances of Sigma–Aldrich [16], and The Good Scents Company [17]. However, the vocabulary used to describe the odorants’ odor is extensive and ambiguous. As a matter of fact, are “citrus odor” and “odor of citrus” referring to the same odor descriptors? [18]. Moreover, there is no agreement about the number of ODs essential to cover the complete range of odor stimuli, which varies from 4 to 146 [19]. Though several teams worked on the different relationships, associations, or similarities between OD, none of them had gained widespread acceptance yet [20,21,22].

In most cases, it is not possible to make a direct link between the OD of the odorants released from an odor source and their perceived odor. This is probably the reason why flavorists, who are experts in creating specific odors from combinations of odor-active raw materials such as molecules, are not using OD but a rather different set of descriptors to organize their practical knowledge acquired along with experience [23]. Indeed, to conceptualize the perception of a specific odor trait of an odor source, further called an odor sensory attribute (OSA), flavorists combine a specific set of odor qualities (OQ). For example, according to an expert flavorist, the OSA “Cherry cooked” is composed of the OQs “Almond”, “Cooked”, “Floral”, “Fruity”, “Green”, “Peel”, and “Spicy”. The OQ may be considered “blocks”, where each block could be composed of several molecules. These molecules have a specific odor that is described with OD (e.g., [24]). In a sense, OQ could be considered as a broad category, related more to odor materials than to molecules. Classifications and flavor wheels, usually dedicated to a specific category of food products such as wine or coffee, have been established and could help to make links between OD and OQ. However, these classifications are highly dependent on the studied databases and/or food products and are hardly reconcilable (e.g., caramel [25]; honey [26]; and wine [27]). For example, whereas the OD “Apple” is classified in the OQ “Fruity” in several databases, the OD “Vanilla” can be found classified in different OQs such as “Spicy”, “Balsamic”, “Warm”, “Wood/Phenolic” or “Caramel/Vanilla” depending on the database. 

To overcome these issues, this paper proposes to provide a structure for the description of odors through the use of an ontological approach to make the link between OQ, the concepts manipulated by experts, and OD, the odor descriptors used to qualify odorants. Therefore, with the help of an expert flavorist, we developed and formalized the Ontology for Odor Perceptual Space (OOPS) to organize the vocabulary of the odor perceptual space and to describe the relationships between the OD and OQ. The aim was to fuse the information expressed by OD in order to formally characterize odors into a conceptual and generic annotation of OQ, namely one not associated with a specific food or odor product. Furthermore, as a proof of concept, we further used the OOPS to predict the odor profiles of two red wines, which is to quantitatively predict the OSA used by a trained sensory panel to describe these wines [28].

## 2. Results and Discussion

### 2.1. The Ontology for Odor Perceptual Space (OOPS)

**Figure 1:**
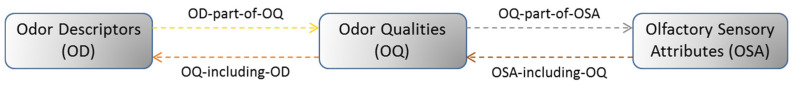
Object properties between the classes OD, OQ, and OSA of the OOPS ontology.

We formalized the Ontology for Odor Perceptual Space (OOPS) as a tuple {C, R, P}, where C corresponded to the three classes OD, OQ, and OSA, with, respectively, 175 sub-classes from database aggregation, 20 sub-classes from expertise collection, and 15 sub-classes from sensory evaluation of the wines; R represented the hierarchical relations among the classes by “is–a” relations; and P, as properties, represented the non-hierarchical associative relations between classes as shown in Figure 1.

**Figure 2:**
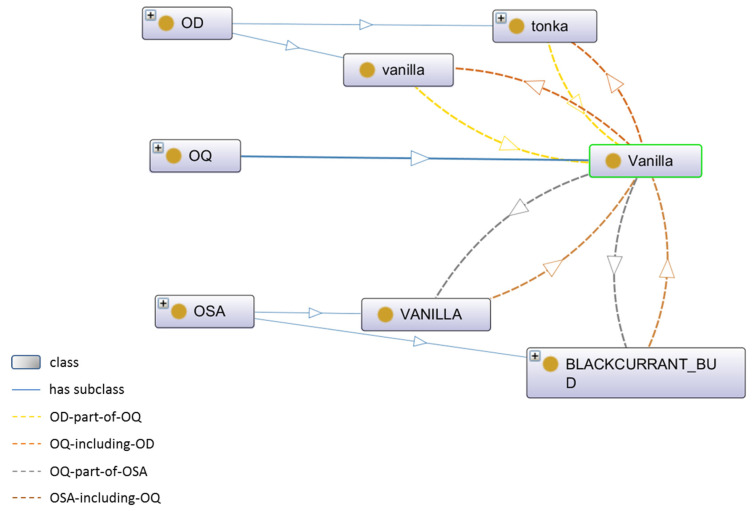
Properties and relationships among the classes OD, OQ, and OSA, considering the OQ “Vanilla”.

Results from the data collection in table forms were implemented in OWL using the software Protégé (open-source ontology editor, version 5.2.0; [29]). This allowed the visualization of the properties among the classes OD, OQ, and OSA; an example is shown in Figure 2 for the OQ “Vanilla”. Such representation highlighted that the OD “vanilla” and “tonka” are parts of the OQ “Vanilla”. Moreover, the OQ “Vanilla” is part of the OSA “VANILLA” and “BLACKCURRANT BUD”. From a practical point of view, these relationships illustrated that an odorant described as “vanilla” or “tonka” is part of the OQ category “Vanilla” and should contribute to the perceptual construction of the odor of Vanilla and Blackcurrant bud, which are OSA in the wine odor context. 

The implementation of the OOPS in OWL conferred the ability to mine the data through queries such as:

- In which OQ is the OD “almond” included? <OQ-including-OD some almond>:“Almond”
- Which OD are parts of the OQ “Almond”? <OD-part-of-OQ some Almond>:“almond”
- In which OSA, the OQ “Almond” is included? <OSA-including-OQ some Almond>: “CHERRY_COOKED”, “CHERRY_FRESH”, “CHERRY_STONE”, “PRUNE”
- Which OQ are parts of the OSA “Prune”? <OQ-part-of-OSA some Prune>: “Almond”, “Cooked”, “Fruity”, “Honey”, “Lactonic”

**Figure 3:**
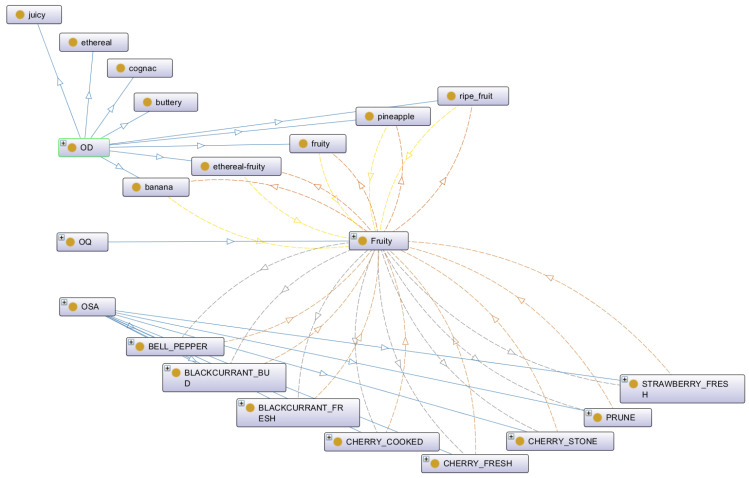
Properties and relationships among the classes OD, OQ, and OSA, considering the OD of the odorant Ethyl butanoate.

Altogether, the OOPS led to the fast visualization of relationships among the three classes (OD, OQ, and OSA) in order to estimate the OQ or OSA profiles of odorants (Figure 3). For example with the odorant ethyl butanoate, described by the OD (Ethyl butanoate) = [(banana, 2); (buttery, 1); (cognac, 1); (ethereal, 1); (ethereal-fruity, 1); (fruity, 2); (juicy, 2); (pineapple, 3); (ripe fruit, 1)], we were able to estimate its contribution to the OQ “Fruity” and then to the OSA “Bell pepper”, “Blackcurrant bud”, “Blackcurrant fresh”, “Cherry cooked”, “Cherry fresh”, “Cherry stone”, “Prune”, and “Strawberry fresh”. 

The intensities of the OD were spread along the relationships between the OD and OQ as well as between the OQ and OSA. The OQ set of Ethyl butanoate was equal to OQ(Ethyl butanoate) = [(Almond, 0); (Cooked, 0); (Cut-grass, 0); (Floral, 0); (Fresh, 0); (Fruity, 9); (Green, 0); (Honey, 0); (Lactony, 0); (Leather, 0); (Peel, 0); (Smoky, 0); (Spicy, 0); (Sulfurous, 0); (Toasty, 0); (Vanilla, 0); (Vegetable, 0); (Violet, 0); (Wine-like, 0); (Woody, 0)], as previously mentioned. Regarding the OSA set, we obtained: OSA (Ethyl butanoate) = [(Bell pepper, 9); (Blackcurrant bud, 9); (Blackcurrant fresh, 9); (Cherry cooked, 9); (Cherry fresh, 9); (Cherry stone, 9); (Cut-grass, 0); (Leather, 0); (Prune, 9); (Smoky, 0); (Strawberry fresh, 9); (Toasty, 0); (Vanilla, 0); (Violet, 0); (Woody, 0)].

### 2.2. Application of the OOPS to Wines

As a proof of concept, we applied the OOPS to establish the OQ and OSA profiles of two wines from their molecular composition. Two wines were selected among the sixteen used to build the ontology: one Pinot noir (PN-A) and one Cabernet Franc (CF-A). We estimated the OQ and OSA sets of each odorant present in the two wines. For a given wine, we summed the OQ and OSA sets of the odorants included in the wine, weighted by their intensities. 

Firstly, we obtained the OQ profiles of the wines PN-A and CF-A, respectively, OQ(PN-A) and OQ(CF-A):

- OQ(PN-A) = [(Almond, 1); (Cooked, 3); (Cut-grass, 2); (Floral, 25); (Fresh, 1); (Fruity, 118); (Green, 12); (Honey, 6); (Lactony, 1); (Leather, 1); (Peel, 4); (Smoky, 24); (Spicy, 10); (Sulfurous, 3); (Toasty, 2); (Vanilla, 4); (Vegetable, 8); (Violet, 0); (Wine-like, 9); (Woody, 5)]
- OQ(CF-A) = [(Almond, 3); (Cooked, 4); (Cut-grass, 1); (Floral, 20); (Fresh, 1); (Fruity, 97); (Green, 15); (Honey, 3); (Lactony, 0); (Leather, 4); (Peel, 4); (Smoky, 20); (Spicy, 1); (Sulfurous, 4); (Toasty, 0); (Vanilla, 0); (Vegetable, 21); (Violet, 0); (Wine-like, 10); (Woody, 4)]

At this step, the two wines were described as "Fruity” wines with “Floral”, “Green”, and “Smoky” notes, and CF-A differed from PN-A with its “Vegetable” note. Then, we obtained the OSA profiles of the wines PN-A and CF-A, respectively: OSA (PN-A) and OSA (CF-A):

- OSA(PN-A) = [(Bell pepper, 51); (Blackcurrant bud, 172); (Blackcurrant fresh, 168); (Cherry cooked, 55); (Cherry fresh, 55); (Cherry stone, 55); (Cut-grass, 2); (Leather, 1); (Prune, 129); (Smoky, 24); (Strawberry fresh, 158); (Toasty, 2); (Vanilla, 4); (Violet, 0); (Woody, 5)]
- OSA(CF-A) = [(Bell pepper, 61); (Blackcurrant bud, 147); (Blackcurrant fresh, 147); (Cherry cooked, 47); (Cherry fresh, 47); (Cherry stone, 47); (Cut-grass, 1); (Leather, 4); (Prune, 107); (Smoky, 20); (Strawberry fresh, 136); (Toasty, 0); (Vanilla, 0); (Violet, 0); (Woody, 4)]

**Figure 4:**
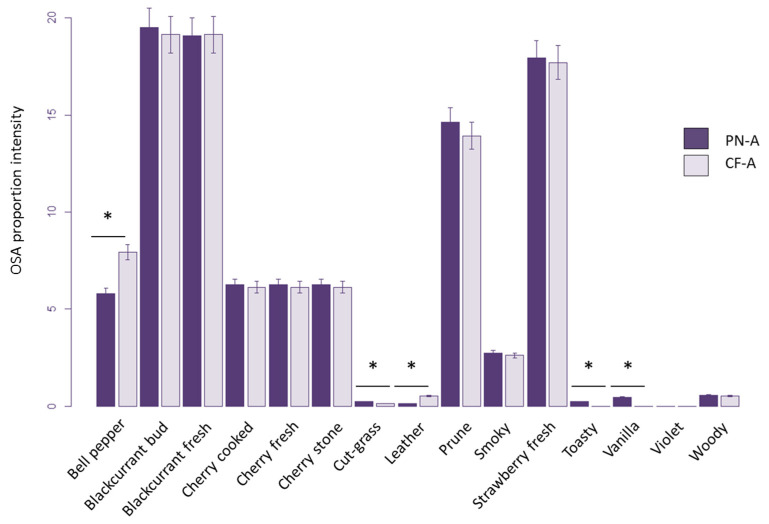
OSA proportions in the PN-A and CF-A wines. Bars display the proportion of OSAs, and wines are indicated by dark (PN-A) or light (CF-A) shading. The horizontal line on the top of the bars indicates a significantly different proportion of OSA between the two wines (* = 5%).

From these OSA sets, we were able to point out differences between the two wines (Figure 4). The PN-A wine was identified as having a higher proportion of intensity of the OSA “Cut-grass”, “Toasty”, and “Vanilla” and a lower proportion of intensity of the OSA “Bell pepper” and “Leather” than the CF-A wine. These results were consistent with the literature because PN and CF wines are described as “Fruity” wines. Moreover, CF wines are usually described as having a “Bell pepper” [30].

According to the sensory profiles of the wines [28], PN-A was perceived as more “Toasty” and “Vanilla” than CF-A, which is also found with the OOPS approach. However, some differences between the wines did not follow their sensory profiles. Indeed, from the sensory evaluation, the CF-A wine was perceived with a higher intensity of the OSA “Cut-grass” and a lower intensity of the OSA “Leather” than PN-A, but from the OOPS approach, we obtained the opposite.

## 3. Materials and Methods

### 3.1. Wines

Villière et al. [28] studied the sensory profiles and the chemical composition in terms of odor-active compounds of sixteen red wines (8 Pinot Noir and 8 Cabernet Franc), varying according to their exemplarity for the grape variety [31]. Sensory profiles resulted in the identification of 15 discriminant OSAs between the wines according to their grape varieties (Table 1). The results of Gas Chromatography–Mass Spectrometry–Olfactometry (GC-MS-O) analyses led to the identification of 46 odorant zones (molecules and mixtures of molecules), which corresponded to 49 identified odorants. Raw data are available in an open-source repository [32].

### 3.2. Elicitation of Odor Qualities (OQ) by Expert Flavorists

Four senior flavorists participated in the expert knowledge collection. The elicitation process was based on a 1 h private guided phone interview. Flavorists were not aware of the studied food matrix in order to collect unbiased data regarding the food product.

The experts monadically received the 15 OSA used in the wines’ sensory profiles (Table 1) and were asked (i) if the OSA was composed of a single OQ or more than one OQ, and (ii) in case the considered OSA was composed of several OQ, to enumerate the OQ that were needed to construct the OSA. Then we aggregated the information of the four flavorists following Equation (1), with OSA being a given odor sensory attribute, Exp1[OQ(OSA)], Exp2[OQ(OSA)], Exp3[OQ(OSA)], and Exp4[OQ(OSA)] being the sets of OQ used to describe an OSA by each of the four experts.

OSA = Exp1[OQ(OSA)]∪Exp2[OQ(OSA)]∪Exp3[OQ(OSA)]∪Exp4[OQ(OSA)](1)

As a result, we obtained a binary matrix made of the 20 OQs elicited (Almond, Cooked, Cut-Grass, Floral, Fresh, Fruity, Green, Honey, Lactony, Leather, Peel, Smoky, Spicy, Sulfurous, Toasty, Vanilla, Vegetable, Violet, Wine-like, and Woody) in rows and the target OSA in columns (Table 2). 

### 3.3. Quantitative Description of the Odorants

We compiled the data from three databases to collect the odor descriptors (OD) of the 49 odorants identified in the wines [28,32]: Arctander’s Handbook (3102 chemicals described by Steffen Arctander himself, [11]), Flavor-Base (commercially available Leffingwell & Associates database, marketed as Flavor-Base Pro © 2010, flavor descriptions collected from many sources over the course of more than 40 years, [14]), and The Good Scents Company (publicly available database, the odor descriptions from one to several sources are listed in the “Organoleptic Properties” section [17]).

We manually extracted the OD from these databases. The words describing the odorants were tokenized. Suffixes (e.g., “like”, “note”), auxiliary verbs (e.g., “has”), and some other words that did not rely on olfactory information (e.g., “powerful”) were discarded. Unlike the analysis of the Arctander database proposed by [19], we kept all the OD into account and we did not combine very similar descriptors (such as Leather/Leathery or Wine/Winey) For instance, the odor of Ethyl butanoate (CAS 105-54-4) was specified in Arctander [11] as “Powerful, ethereal-fruity odor suggestive of banana and pineapple, and very diffusive”. Following our methodology, these annotations resulted in the set of ODs: “ethereal-fruity”, “banana” and “pineapple”. 

Then we created the OD database by aggregating the information from the three databases following Equation (2), M being a given odorant, Arct[OD(M)], FlavorBase[OD(M)], and Goodscent[OD(M)] being the sets of ODs of the odorant M by the Arctander, Flavor-Base and Goodscent databases. We ended up with 175 different ODs for the 49 odorants.

OD database(M) = Arct[OD(M)]∪FlavorBase[OD(M)]∪Goodscent[OD(M)](2)

For a given odorant, a description was thus provided by the OD database as a set of terms, in which each term may be associated with an “intensity”. We defined this intensity as the number of citations of the same OD for a given odorant across the databases: the higher the number of citations, the more “intense” the smell related to this OD was expected for the odorant. As an example, the odorant description of Ethyl butanoate was {ethereal-fruity; banana; pineapple} by Arctander [11], {ethereal; fruity; buttery; pineapple; banana; ripe fruit; juicy} by Flavor-base [14], and {fruity; juicy; pineapple; cognac} by GoodScents [17]. The resulting quantitative description of Ethyl butanoate in the OD database was the following: OD(Ethyl butanoate) = [(banana, 2); (buttery, 1); (cognac, 1); (ethereal, 1); (ethereal-fruity, 1); (fruity, 2); (juicy, 2); (pineapple, 3); (ripe fruit, 1)].

### 3.4. Relationships between Odor Descriptors (OD) and Odor Qualities (OQ)

The correspondence between an OD and one or more OQ has been obtained thanks to the expertise of a junior flavorist. This expert was not one of the four flavorists previously interviewed for OQ elicitation. The methodology used to obtain the relationships was based on a “check-all-that-apply” (CATA) questionnaire [33]. The CATA list consisted of the 20 OQ defined by the experts during the elicitation step (see Section 2.2. above). For each OD in the OD database, the flavorist was asked if the OD supported none, one, or several OQ. For instance, for the OD “Apple”, the flavorist was asked to tick all the OQs that correspond, e.g., “Fruity”.

We obtained a binary matrix with the OQ in columns and the OD in rows (Table 3). These results allowed us to translate each OD set into OQ sets. For example, with Ethyl butanoate, described as OD(Ethyl butanoate) = [(banana, 2); (buttery, 1); (cognac, 1); (ethereal, 1); (ethereal-fruity, 1); (fruity, 2); (juicy, 2); (pineapple, 3); (ripe fruit, 1)]. We could then assume that the OQ set of Ethyl butanoate was the following: OQ(Ethyl butanoate) = [(Almond, 0); (Cooked, 0); (Cut-grass, 0); (Floral, 0); (Fresh, 0); (Fruity, 9); (Green, 0); (Honey, 0); (Lactony, 0); (Leather, 0); (Peel, 0); (Smoky, 0); (Spicy, 0); (Sulfurous, 0); (Toasty, 0); (Vanilla, 0); (Vegetable, 0); (Violet, 0); (Wine-like, 0); (Woody, 0)].

## 4. Conclusions and Future Work

In this paper, we presented the building of the OOPS, the Ontology for Odor Perceptual Space, designed for fixing the vocabulary of the odor perceptual space and the relationships between the different terms involved: OD, OQ, and OSA. The genericity of the OOPS was achieved by integrating the flavorist’s expertise. An example of the application of the OOPS to a food product was presented with the odorant composition of two red wines to estimate their OQ and OSA profiles. We were able to obtain a good prediction of the OQ and OSA profiles.

This work, following a semantic approach, will provide a standard tool for communication among experts to increase knowledge sharing and can be helpful in training sensory panels for odor profiling. Therefore, this ontology might be used to establish sensory profiles of food products based on their chemical composition. Because of the genericity of the tool, the OOPS will be available for studying various food products. 

However, we would like to highlight that this approach has several ways of improvement. We should keep in mind that the perception of an odorant mixture is not the simple sum of each odorant’s odor. Non-linear combinations among the OD, OQ, and OSA could then be developed from the knowledge we collected and formalized. In addition, the intensity or concentration of odorants might be integrated into the OOPS approach to get a more precise balance of the OD sets that further impact OQ and OSA profile prediction.

Finally, one advantage of ontology formalization is that data could be further enriched and/or modified to adapt to domain changes or new usages. Indeed, OD or OQ may become outdated and may be incomprehensible to subjects from different cultural backgrounds or non-native English speakers. One of the following works will be to increase the data and knowledge embedded in the OOPS to allow for more complete and accurate predictions.

## Figures and Tables

**Table 1:** List of the 15 odor sensory attributes (OSA).

Bell pepper
Blackcurrant bud
Blackcurrant fresh
Cherry cooked
Cherry fresh
Cherry stone
Cut-grass
Leather
Prune
Smoky
Strawberry fresh
Toasty
Vanilla
Violet
Woody

**Table 2:** Link between the 20 OQ (rows) and the 15 OSA (columns), represented as a binary matrix. The value 1 indicates that the OQ was part of the composition of the OSA.

OQ	Bell pepper	Blackcurrant Bud	Blackcurrant Fresh	Cherry Cooked	Cherry Fresh	Cherry Stone	Cut-Grass	Leather	Prune	Smoky	Strawberry Fresh	Toasty	Vanilla	Violet	Woody
Almond				1	1	1			1						
Cooked				1	1	1			1		1				
Cut-grass							1								
Floral	1	1	1	1	1	1					1				
Fresh	1	1	1												
Fruity		1	1						1		1				
Green	1	1	1	1	1	1					1				
Honey									1						
Lactoniy									1						
Leather								1							
Peel				1	1	1									
Smoky										1					
Spicy				1	1	1									
Sulfurous	1	1	1												
Toasty	1											1			
Vanilla		1											1		
Vegetable	1														
Violet															
Wine-like		1	1												
Woody															1

**Table 3:** Link between the nine ODs of Ethyl butanoate (rows) and the 20 OQs (columns), represented as a binary matrix. The intensity of each OD is specified in the second column.

OD	Intensity	Almond	Cooked	Cut-grass	Floral	Fresh	Fruity	Green	Honey	Lactonic	Leather	Peel	Smoky	Spicy	Sulfurous	Toasty	Vanilla	Vegetable	Violet	Wine-like	Woody
banana	2	0	0	0	0	0	1	0	0	0	0	0	0	0	0	0	0	0	0	0	0
buttery	1	0	0	0	0	0	0	0	0	0	0	0	0	0	0	0	0	0	0	0	0
cognac	1	0	0	0	0	0	0	0	0	0	0	0	0	0	0	0	0	0	0	0	0
ethereal	1	0	0	0	0	0	0	0	0	0	0	0	0	0	0	0	0	0	0	0	0
ethereal-fruity	1	0	0	0	0	0	1	0	0	0	0	0	0	0	0	0	0	0	0	0	0
fruity	2	0	0	0	0	0	1	0	0	0	0	0	0	0	0	0	0	0	0	0	0
juicy	2	0	0	0	0	0	0	0	0	0	0	0	0	0	0	0	0	0	0	0	0
pineapple	3	0	0	0	0	0	1	0	0	0	0	0	0	0	0	0	0	0	0	0	0
ripe fruit	1	0	0	0	0	0	1	0	0	0	0	0	0	0	0	0	0	0	0	0	0

## Data Availability

The data presented in this study are available upon request from the corresponding author.

## References

[1] Dubois D., Rouby C., Rouby C., Schaal B., Dubois D., Gervais R., Holley A. (2002). Names and categories for odors: The veridical label. Olfaction, Taste and Cognition.

[2] Thomas-Danguin T., Sinding C., Romagny S., El Mountassir F., Atanasova B., Le Berre E., Le Bon A.-M., Coureaud G. (2014). The perception of odor objects in everyday life: A review on the processing of odor mixtures. Front. Psychol..

[3] Cain W.S., Drexler M. (1974). Scope and evaluation of odor counteraction and masking. Ann. N. Y. Acad. Sci..

[4] Ishii A., Roudnitzky N., Beno N., Bensafi M., Hummel T., Rouby C., Thomas-Danguin T. (2008). Synergy and masking in odor mixtures: An electrophysiological study of orthonasal V.S retronasal perception. Chem. Senses.

[5] Ferreira V. (2012). Revisiting psychophysical work on the quantitative and qualitative odour properties of simple odour mixtures: A flavour chemistry view. Part 1: Intensity and detectability. A review. Flavour Fragr. J..

[6] Tempere S., Hamtat M.-L., de Revel G., Sicard G. (2016). Comparison of the ability of wine experts and novices to identify odorant signals: A new insight in wine expertise. Aust. J. Grape Wine Res..

[7] Ferreira V. (2012). Revisiting psychophysical work on the quantitative and qualitative odour properties of simple odour mixtures: A flavour chemistry view. Part 2: Qualitative aspects. A review. Flavour Fragr. J..

[8] Jinks A., Laing D.G. (2001). The analysis of odor mixtures by humans: Evidence for a configurational process. Physiol. Behav..

[9] Romagny S., Coureaud G., Thomas-Danguin T. (2018). Key odorants or key associations? Insights into elemental and configural odour processing. Flavour Fragr. J..

[10] Le Berre E., Béno N., Ishii A., Chabanet C., Etievant P., Thomas-Danguin T. (2008). Just noticeable differences in component concentrations modify the odor quality of a blending mixture. Chem. Senses.

[11] Arctander S. (1969). Perfume and Flavor Chemicals.

[12] Dravnieks A. (1985). Atlas of Odor Character Profiles.

[13] Burdock G.A. (2010). Fenaroli’s Handbook of Flavor Ingredients.

[14] Leffingwell & Associates Flavor-Base.

[15] Acree T., Arn H. Flavornet. https://www.flavornet.org/.

[16] Sigma-Aldrich. https://www.sigmaaldrich.com/FR/en/products/chemistry-and-biochemicals/flavors-and-fragrances.

[17] Luebke W. The Good Scents Company. http://www.thegoodscentscompany.com/index.html.

[18] Barkat-Defradas M., Motte-Florac E., Barkat-Defradas M., Motte-Florac E. (2016). Verbalization of olfactory perception. Words for Odours: Language Skills and Cultural Insights.

[19] Chastrette M., Rouby C., Schaal B., Dubois D., Gervais R., Holley A. (2002). Classification of odors and structure-odor relationships. Olfaction, Taste and Cognition.

[20] Chastrette M., Elmouaffek A., Sauvegrain P. (1988). A multidimensional statistical study of similarities between 74 notes used in perfumery. Chem. Senses.

[21] Zarzo M., Stanton D.T. (2006). Identification of latent variables in a semantic odor profile database using principal component analysis. Chem. Senses.

[22] Kumar R., Kaur R., Auffarth B., Bhondekar A.P. (2015). Understanding the odour spaces: A step towards solving olfactory stimulus-percept problem. PLoS ONE.

[23] Langlois J., Dacremont C., Peyron D., Valentin D., Dubois D. (2011). Lexicon and types of discourse in wine expertise: The case of vin de garde. Food Qual. Prefer..

[24] Jaubert J.N., Gordon G., Dore J.-C. (1987). Une organisation du champ des odeurs. Première partie: Recherche de critères objectifs. Parfum. Cosmétiques Arômes.

[25] Paravisini L., Septier C., Moretton C., Nigay H., Arvisenet G., Guichard E., Dacremont C. (2014). Caramel odor: Contribution of volatile compounds according to their odor qualities to caramel typicality. Food Res. Int..

[26] International Honey Commission (IHC). http://www.ihc-platform.net/reports.html.

[27] Noble A.C., Arnold R.A., Buechsenstein J., Leach E.J., Schmidt J.O., Stern P.M. (1987). Modification of a Standardized System of Wine Aroma Terminology. Am. J. Enol. Vitic..

[28] Villière A., Symoneaux R., Roche A., Eslami A., Perrot N., Le Fur Y., Prost C., Courcoux P., Vigneau E., Thomas-Danguin T. (2019). Characterization of the flavor of two red wine varieties using sensory descriptive analysis, volatile organic compounds quantitative analysis by GC-MS and odorant composition by GC-MS-O. Data Brief.

[29] Musen M.A. (2015). The Protégé project: A look back and a look forward. AI Matters.

[30] Lawrence G., Symoneaux R., Maitre I., Brossaud F., Maestrojuan M., Mehinagic E. (2013). Using the free comments method for sensory characterisation of Cabernet Franc wines: Comparison with classical profiling in a professional context. Food Qual. Prefer..

[31] Loison A., Symoneaux R., Deneulin P., Thomas-Danguin T., Fant C., Guérin L., Le Fur Y. (2015). Exemplarity measurement and estimation of the level of interjudge agreement for two categories of French red wines. Food Qual. Prefer..

[32] Villière A., Symoneaux R., Roche A., Eslami A., Perrot N., Le Fur Y., Prost C., Courcoux P., Vigneau E., Thomas-Danguin T. (2018). Dataset on the characterization of the flavor of two red wine varieties using sensory descriptive analysis, volatile organic compounds quantitative analysis by GC-MS and odorant composition by GC-MS-O [Data set]. Zenodo.

[33] Dooley L., Lee Y.S., Meullenet J.F. (2010). The application of check-all-that-apply (CATA) consumer profiling to preference mapping of vanilla ice cream and its comparison to classical external preference mapping. Food Qual. Prefer..

